# Supplementary material for: Structural insights into cardiolipin replacement by phosphatidylglycerol in a cardiolipin-lacking yeast respiratory supercomplex
Source: Nat Commun. 2023 May 15;14:2783. doi: 10.1038/s41467-023-38441-5 (PMC10185535; doi:10.1038/s41467-023-38441-5)
Supplement: Supplementary file 1 — Supplementary Information [file 41467_2023_38441_MOESM1_ESM.pdf]

Supplementary Figures and Tables for the following manuscript

Structural insights into cardiolipin replacement by phosphatidylglycerol in a cardiolipin-lacking yeast respiratory supercomplex using cryo-EM

Corey F. Hryc[1], Venkata K. P. S. Mallampalli[2], Evgeniy I. Bovshik[1], Stavros Azinas[1], Guizhen Fan[2], Irina I. Serysheva[2], Genevieve C. Sparagna[3], Matthew L. Baker[2*], Eugenia Mileykovskaya[1]*, William Dowhan[4]*

[1]Department of Biochemistry and Molecular Biology, McGovern Medical School at The University of Texas Health Science Center Houston, Texas, USA. [2]Department of Biochemistry and Molecular Biology, Structural Biology Imaging Center, McGovern Medical School at The University of Texas Health Science Center Houston, Texas, USA. [3]Department of Medicine, Division of Cardiology, University of Colorado Anschutz Medical Campus, Aurora, Colorado, USA. [4]Department of Biochemistry and Molecular Biology, Center for Membrane Biology, McGovern Medical School at The University of Texas Health Science Center Houston, Texas USA. *mailto:william.dowhan@uth.tmc.edu; mailto:eugenia.Mileykovskaya@uth.tmc.edu; mailto:matthew.l.baker@uth.tmc.edu

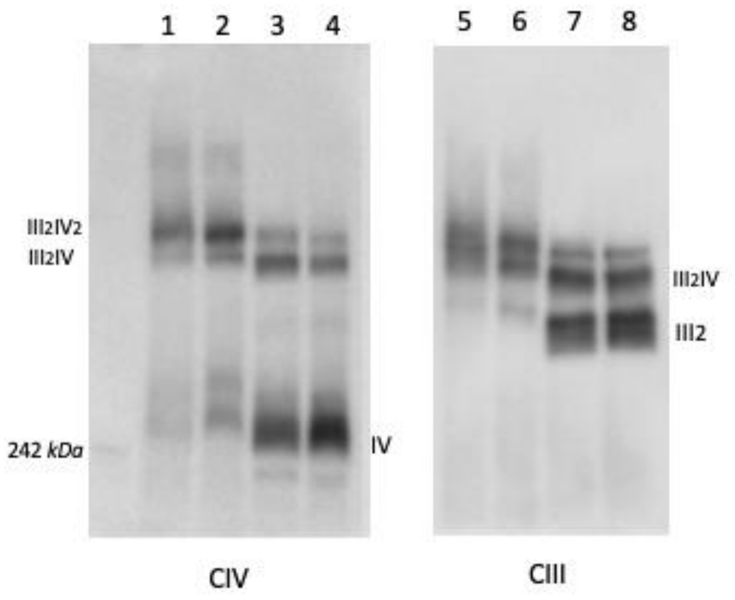

Supplementary Figure 1. Western blot analysis of the digitonin extracts from WT (wild type) and CRD1Δ yeast mitochondria probed with primary antibody to CIV (left) and CIII (right). 10 µg of total protein loaded per well (See methods for details). Displayed is a single experiment as a function of digitonin/protein ratios. However, numerous experiments over several years have been performed using a ratio of 2.5:1 obtaining the same results.

1 – WT 2.5:1 digitonin/protein ratio
2 – WT 6:1 digitonin/protein ratio
3 – CRD1Δ 2.5:1 digitonin/protein ratio
4 – CRD1Δ 6:1 digitonin/protein ratio
5 – WT 2.5:1 digitonin/protein ratio
6 – WT 6:1 digitonin/protein ratio
7 – CRD1Δ 2.5:1 digitonin/protein ratio
8 – CRD1Δ 6:1 digitonin/protein ratio

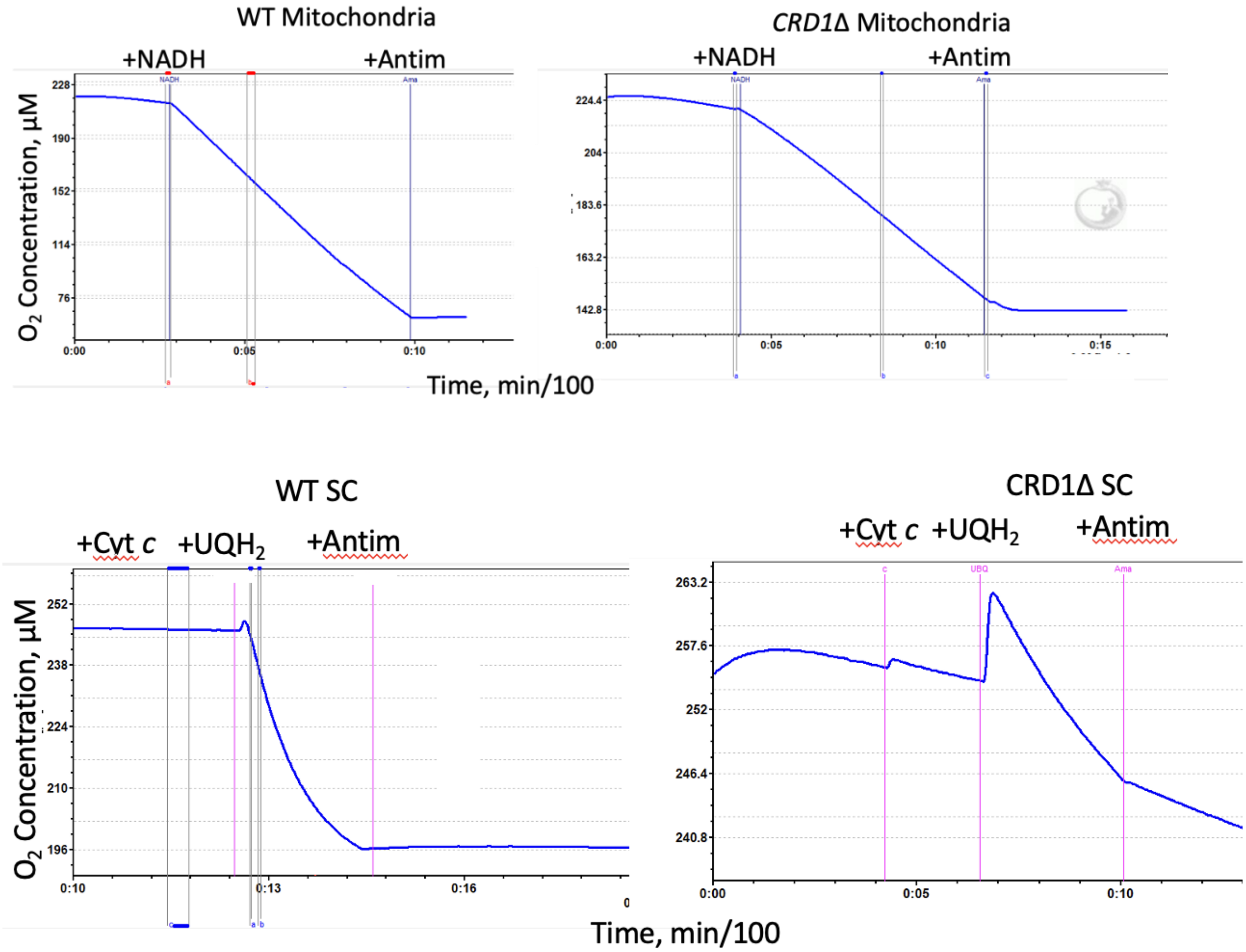

Supplementary Figure 2. Representative oxygen consumption graphs. See Methods for details. Chamber volume was 2 ml and protein concentration was in mg/ml. In the text O_2 consumption is converted to µmoles O_2/min/mg. Oxygen consumption was performed 3 times using a single sample except for ΔCRD1 SC sample, which was done once. Average values and standard deviations are reported in the text. Point of addition of Cyt *c* (cytochrome *c*) UQH_2 (reduced decylubiquinone) and Antim (antimycin A) addition shown. Calculation of oxygen consumption is in Supplementary Table 3. The software package associated with the Oxygraph-2k high resolution respirometry system (Oroboros Instrument) is DatLab-version 7.3.0.3. Source data for all assays used to calculate final results are provided in the Source Data file.

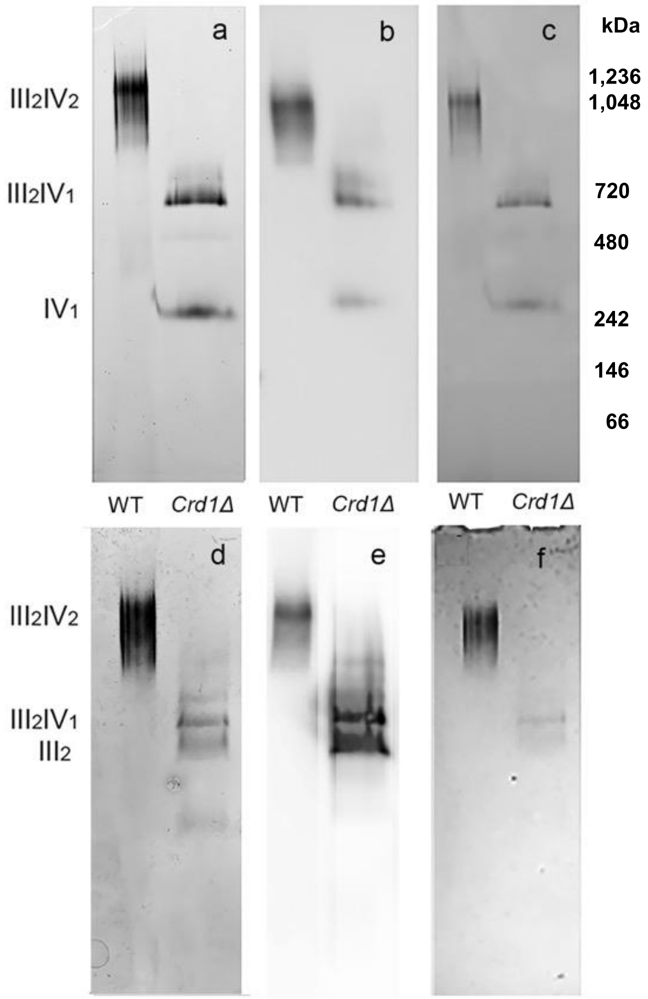

Supplementary Figure 3. Electrophoretic analyses of the respiratory SCs isolated from the WT and CRD1Δ strains. Samples used in these analyses were adjacent to fractions containing the highest purified CRD1Δ SCs and therefore contained free CIII and CIV. Samples were subjected to BN-PAGE (**a-c**) or CN-PAGE (**d-f**). Gels **a** and **d** were stained with Coomassie blue. Gels **b** or **e** were subjected to Western blot analysis using primary antibody to CIV (**b**) or CIII (**e**), respectively. Activity was determined for CIV in gel **c** and for CIII in gel **f**. See Methods for gel electrophoresis, Western blot analysis and in-gel enzyme activity. For Coomassie blue staining and CIV and CIII in-gel activity 1.4 μg of WT SC and 2 μg of CRD1Δ SC were loaded on the respective gels. For Western blot analysis 0.4 μg of WT and 0.5 μg of CRD1Δ SC were loaded on the respective gels. See Supplementary Fig. 18 for placement of MW standards. All gels and blots only have loading wells and very bottoms removed. These experiments were performed 3 to 5 times with similar results.

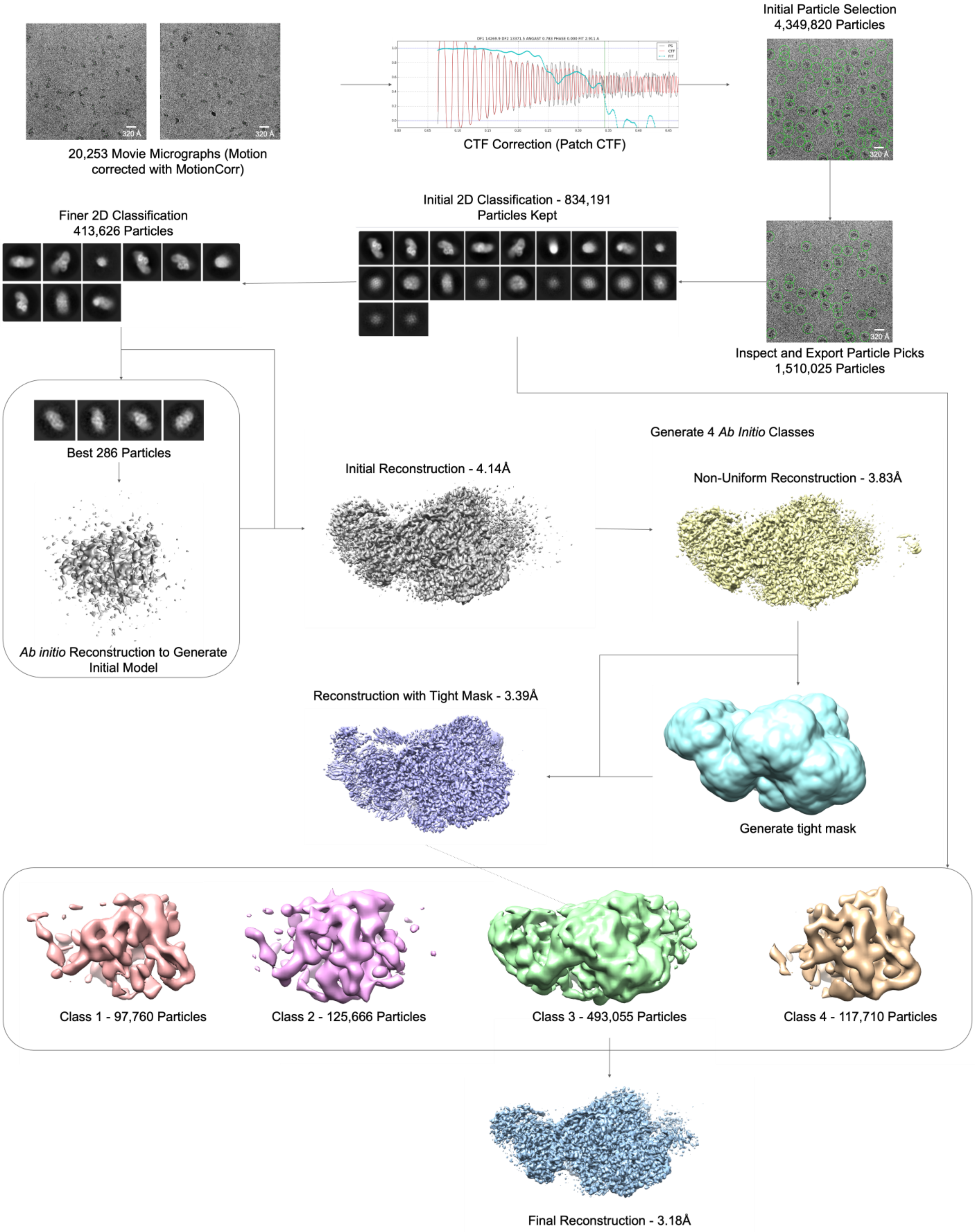

Supplementary Figure 4. WT SC tetramer workflow pathway. Note that the reconstruction with the tight mask (3.39 Å) matches the same data as Class 3 (displayed with a dashed line), however Class 3 has a few more particles due to classification of a larger dataset.

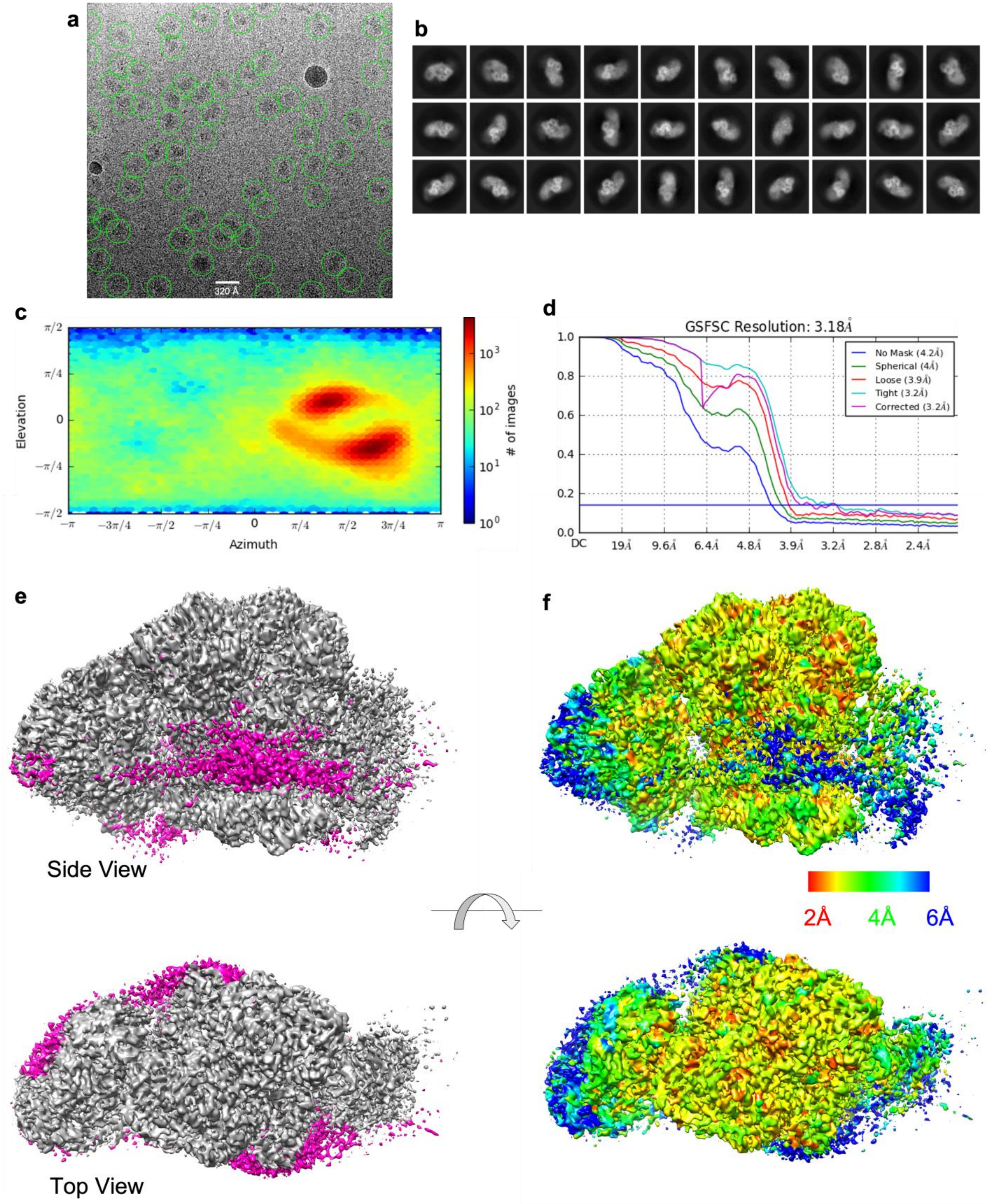

Supplementary Figure 5. Cryo-EM map details of the WT SC. **a.** An example (out of scores of similar views) micrograph movie of the SC tetramer (See Methods for additional details). **b**. Representative 2D class averages. **c**. This abundance of top views can be further displayed by assessing orientation determination. **d**. Fourier shell correlation plot revealing the resolution with various masks applied. **e**. The final cryo-EM density map (side and top views) is show with the excess detergent region (noise) in magenta. **f**. Local resolution plot was mapped onto the structure (shown with a side and top view).

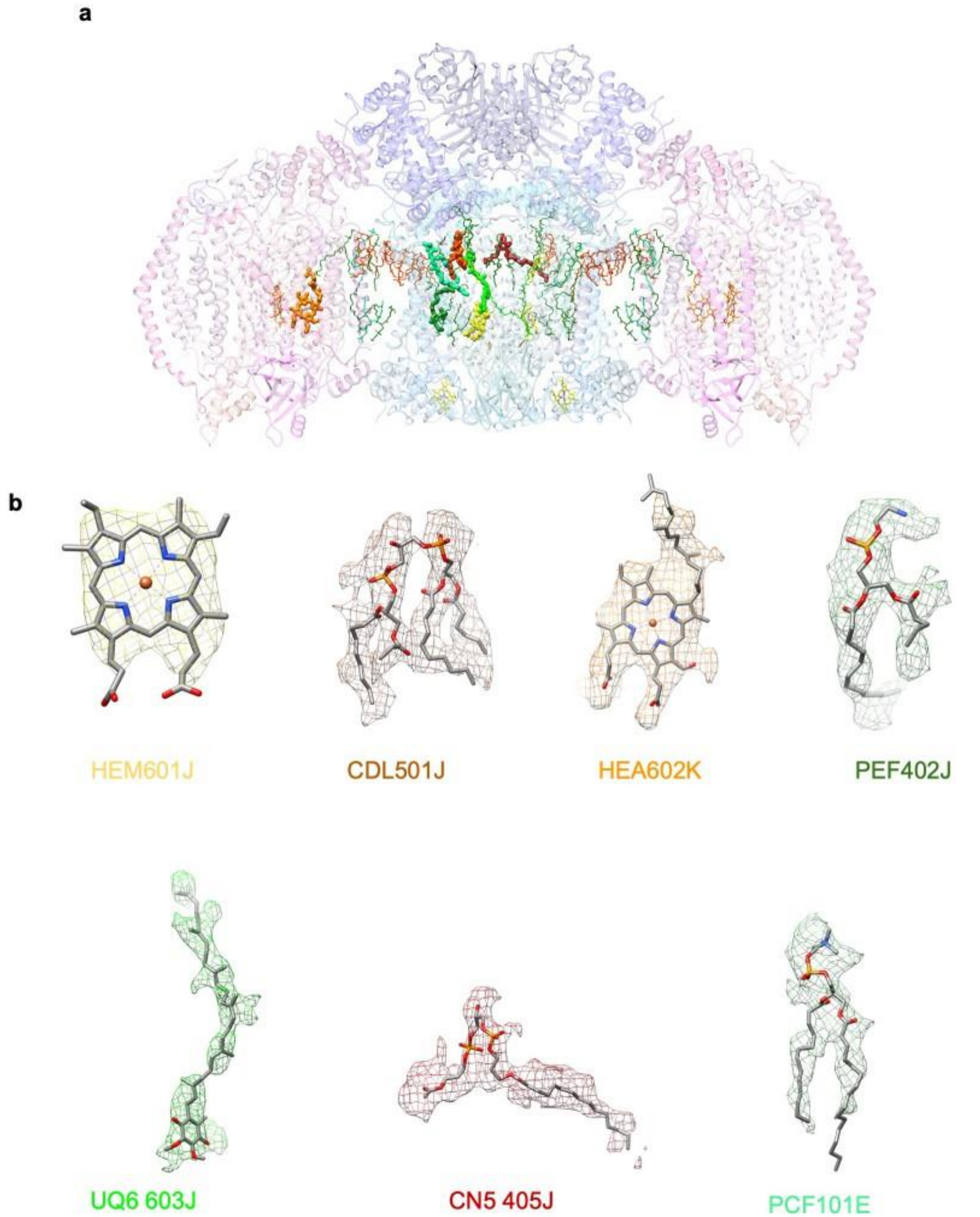

Supplementary Figure 6. Gallery of WT SC tetramer ligands colored by type. **a**. The tetramer model is shown at the top with ligand densities colored for representative views. **b**. Representative ligands (from panel **a**) are shown zoomed-in with the model and their isolated densities from the 3.2 Å map.

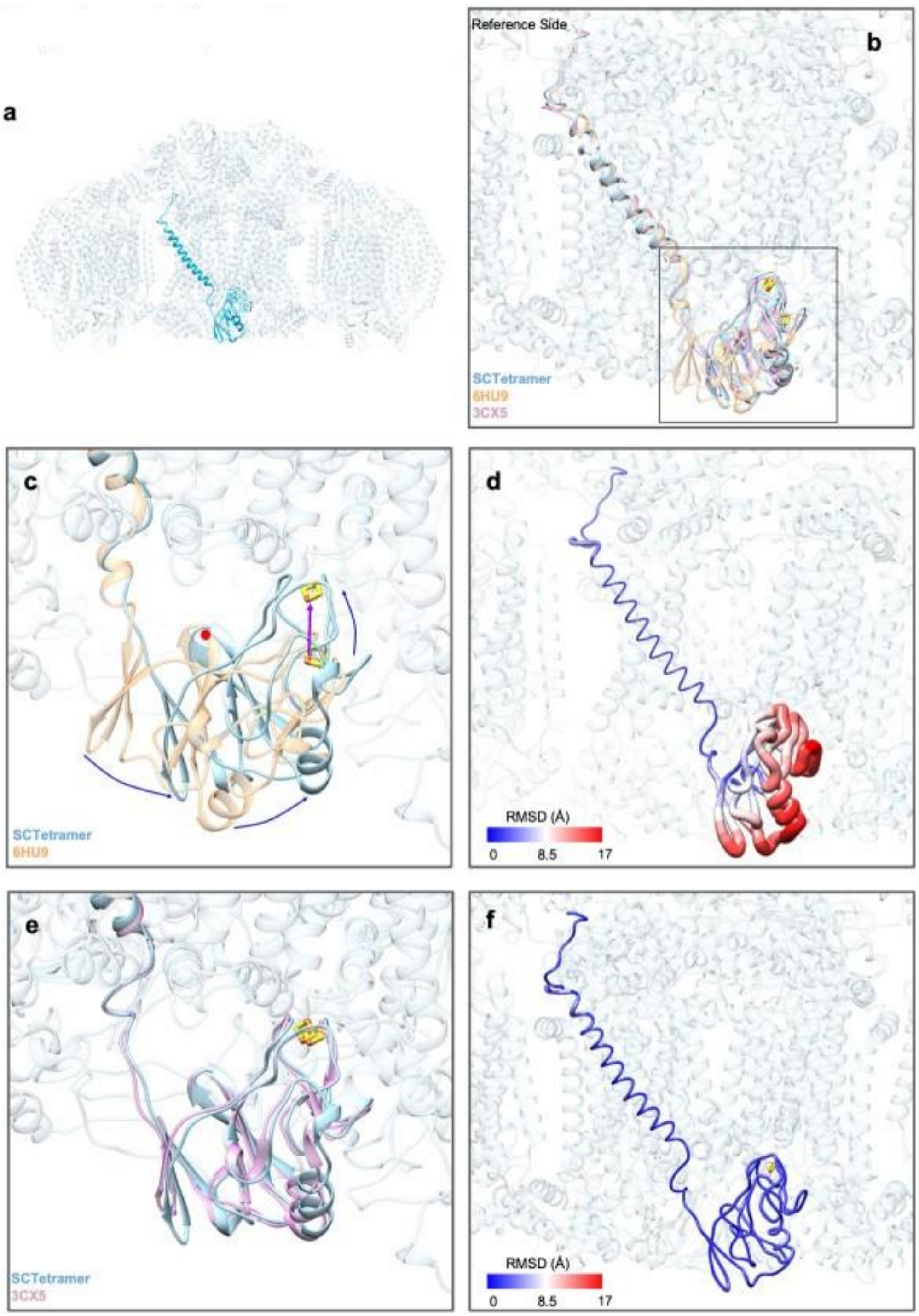

Supplementary Figure 7. Model variation of Rip1 on the reference-side for the WT tetrameric SC structure. **a**. Rip1 position (highlighted in the darker sky blue) of the tetrameric SC with respect to other subunits in the complex. **b**. Comparisons to known structures 6HU9 (shown in light orange) and 3CX5 (shown in light pink) were then done for Rip1. **c**. A zoomed-in view from panel **b** reveals the angular variation of Rip1 on the p-side of the SC compared to 6HU9. The blue arrows highlight the deviation of the loops and the helix, while the red dot represents the possible pivot point that the domain seems to be rotated around. **d**. Root-mean-square deviation (RMSD) between Rip1 of our WT tetrameric SC model and Rip1 of 6HU9. RMSD was computed by first aligning and fitting the density maps and then subsequently the models. Then Chimera's "Match -> Align" tool was used to align the sequence and evaluate the distance between C-alpha pairs. **e**. A zoomed-in view from panel **b** reveals the small variation of Rip1 on the p-side of the SC when compared to 3CX5. **f**. RMSD between Rip1 of the tetrameric SC model and Rip1 of 3CX5 CIII. RMSD was computed the same as the RMSD in panel **d**.

a

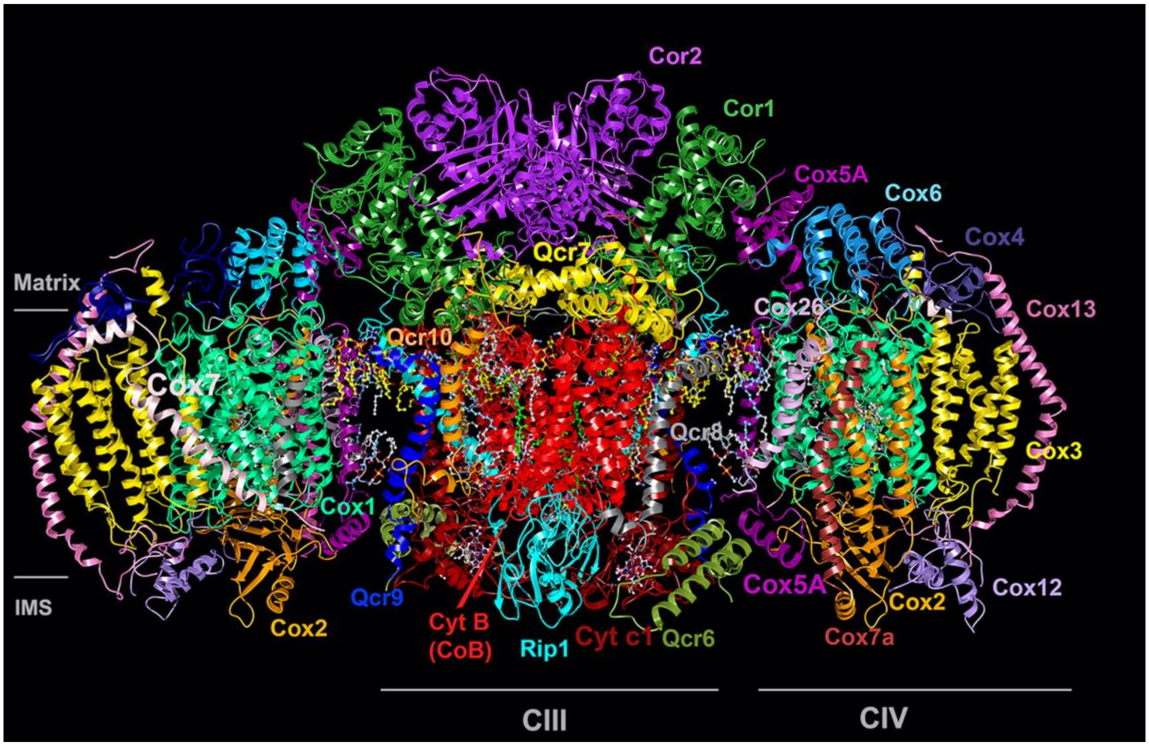

b

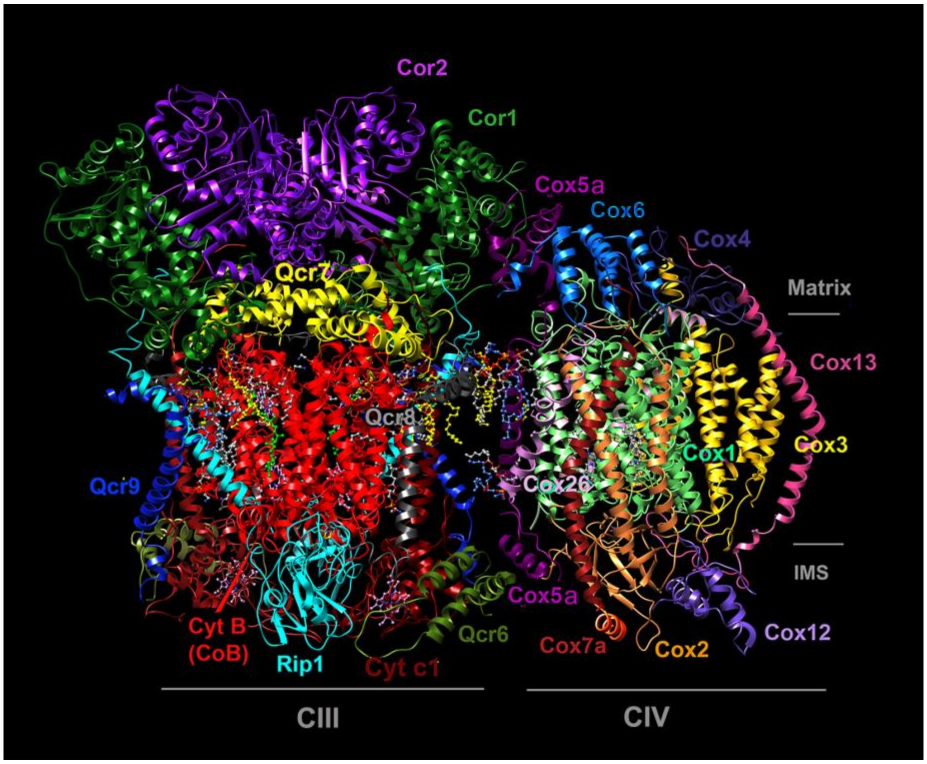

Supplementary Figure 8. Subunit composition and positions in the WT (**a**) and CRD1Δ (**b**) SC structures. Each subunit color and name are indicated in the same color.

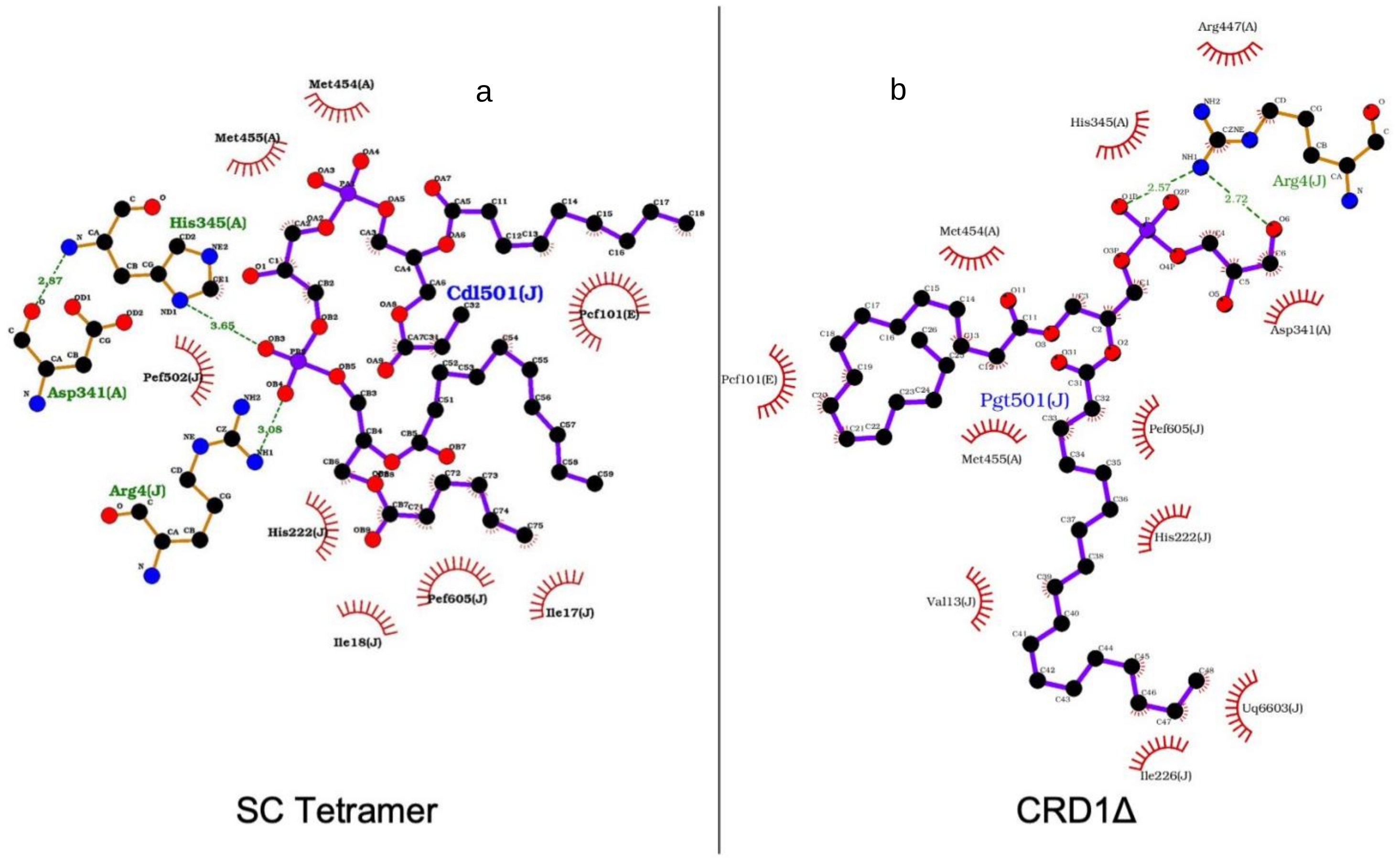

Supplementary Figure 9. LigPlot+ images of CDL 501J (**a**) and PGT 501J (**b**). Hydrogen bonds – green dashes; hydrophobic interactions – red arcs; carbon – black; oxygen – red; nitrogen – blue; ligand bonds – purple; protein bonds – orange.

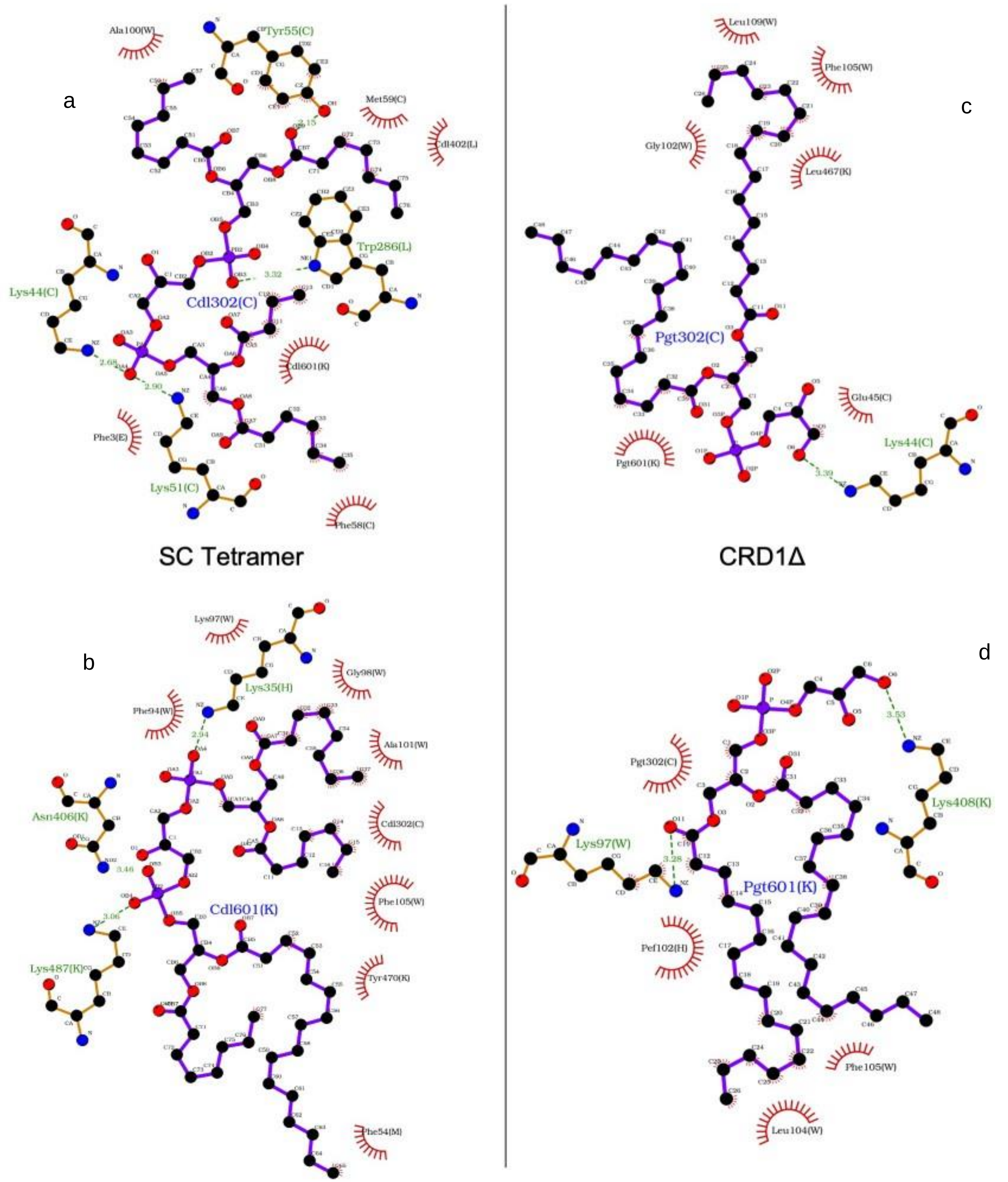

Supplementary Figure 10. LigPlot+ Images of (**a**) CDL 302C; (**b**) CDL 601K; (**c**) PGT 302C; (**d**) PGT601K .Hydrogen bonds – green dashes; hydrophobic interactions – red arcs; carbon – black; oxygen – red; nitrogen – blue; ligand bonds – purple; protein bonds – orange.

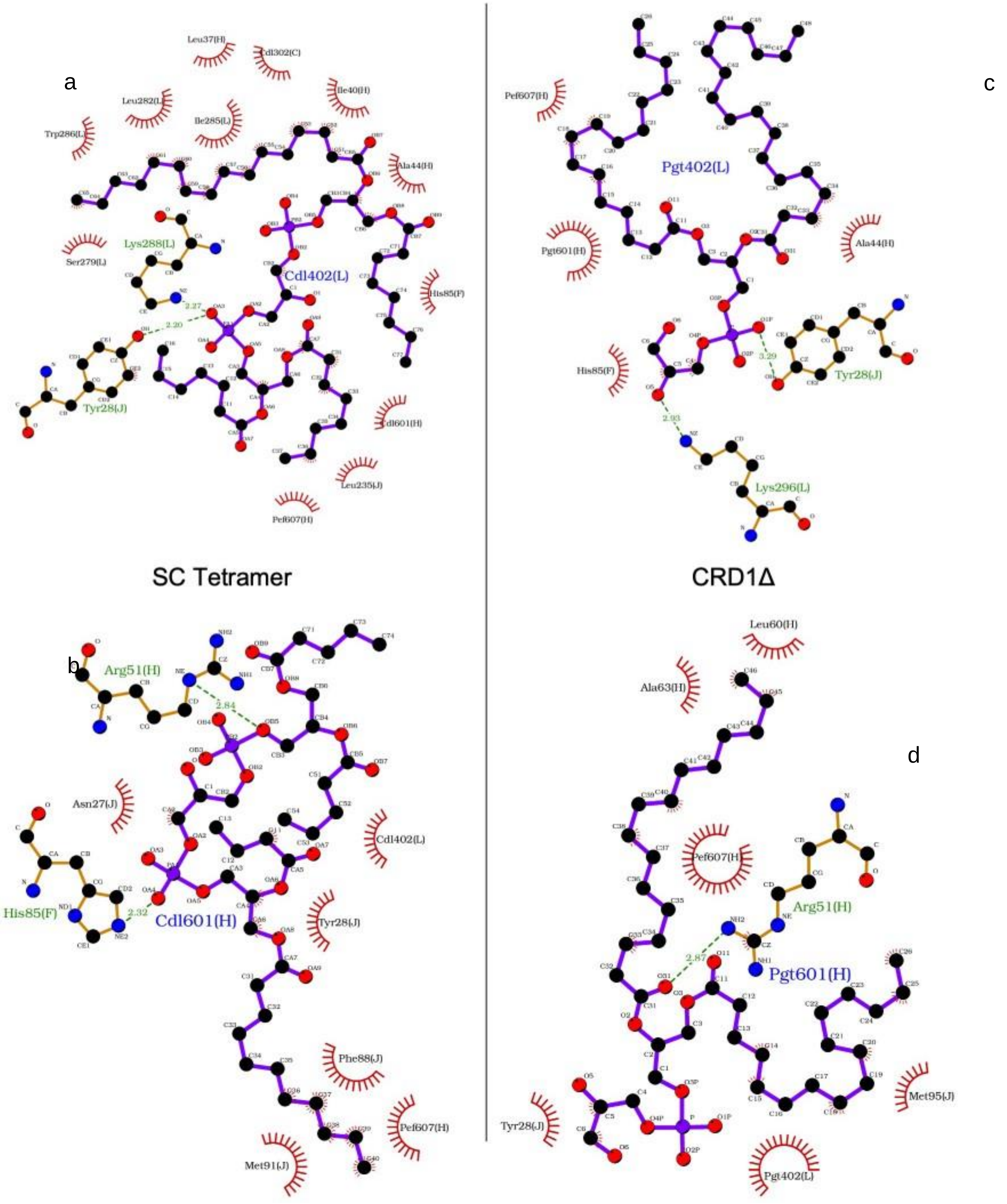

Supplementary Figure 11. LigPlot+ Images of (**a**) CDL402L; (**b**) CDL601H; (**c**) PGT402L; (**d**) PGT601H. Hydrogen bonds – green dashes; hydrophobic interactions – red arcs; carbon – black; oxygen – red; nitrogen – blue; ligand bonds – purple; protein bonds – orange.

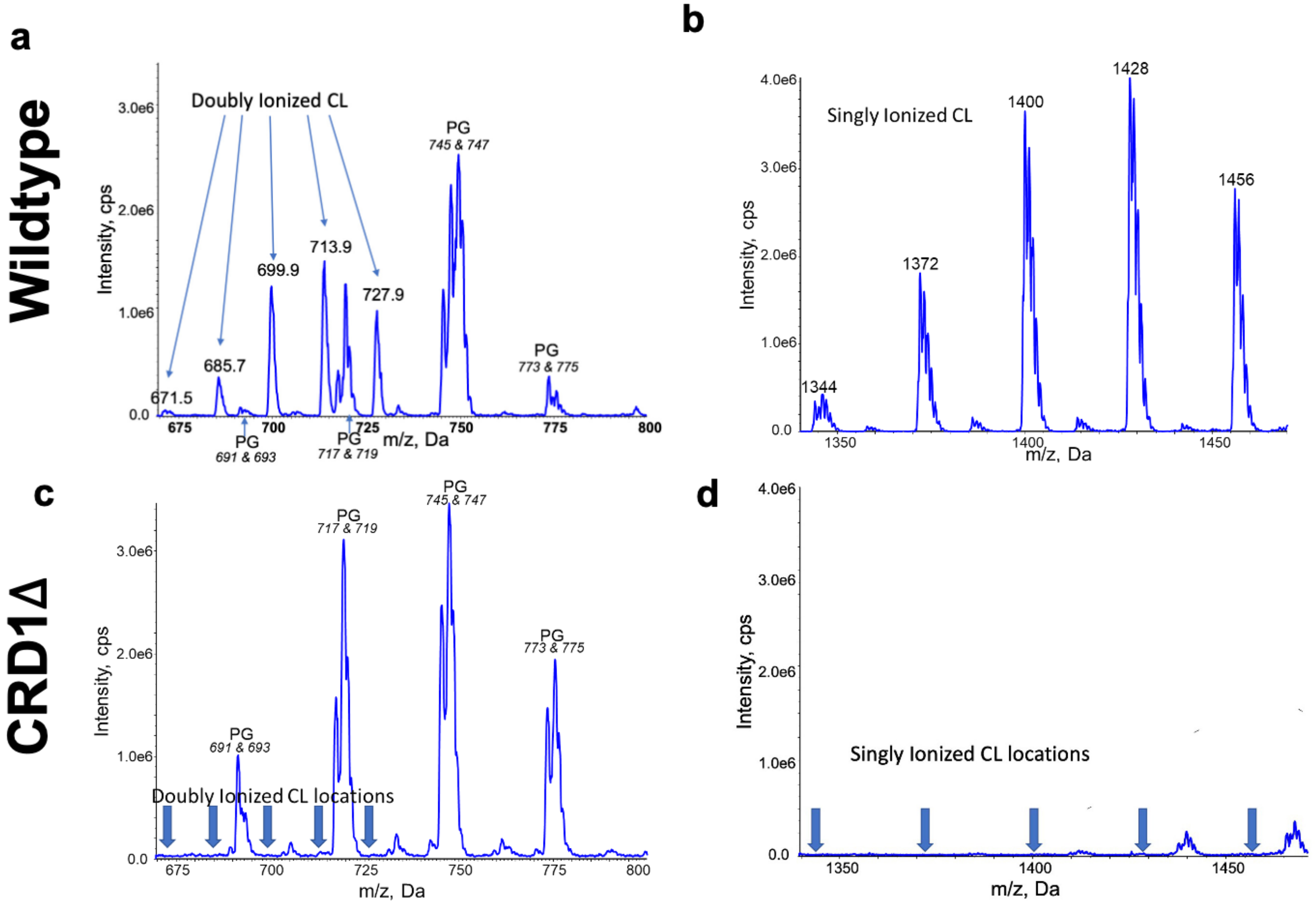

Supplementary Figure 12. Mass spectra for CL and PG in isolated mitoplasts from the WT (**a**, **b**) and *CRD1*Δ mutant (**c**, **d**) yeast strains. **a**. PG and doubly ionized CL are indicated for the WT mitoplasts. **b**. Singly ionized CL peaks in the WT mitoplasts. **c**. PG peaks in the mutant are indicated and thick arrows indicate where doubly ionized CL peaks would be located. **d**. Arrows indicate where singly ionized CL peaks would be located. Sum of each peak is presented in Supplementary Table 5. Source data are provided in the Source Data file.

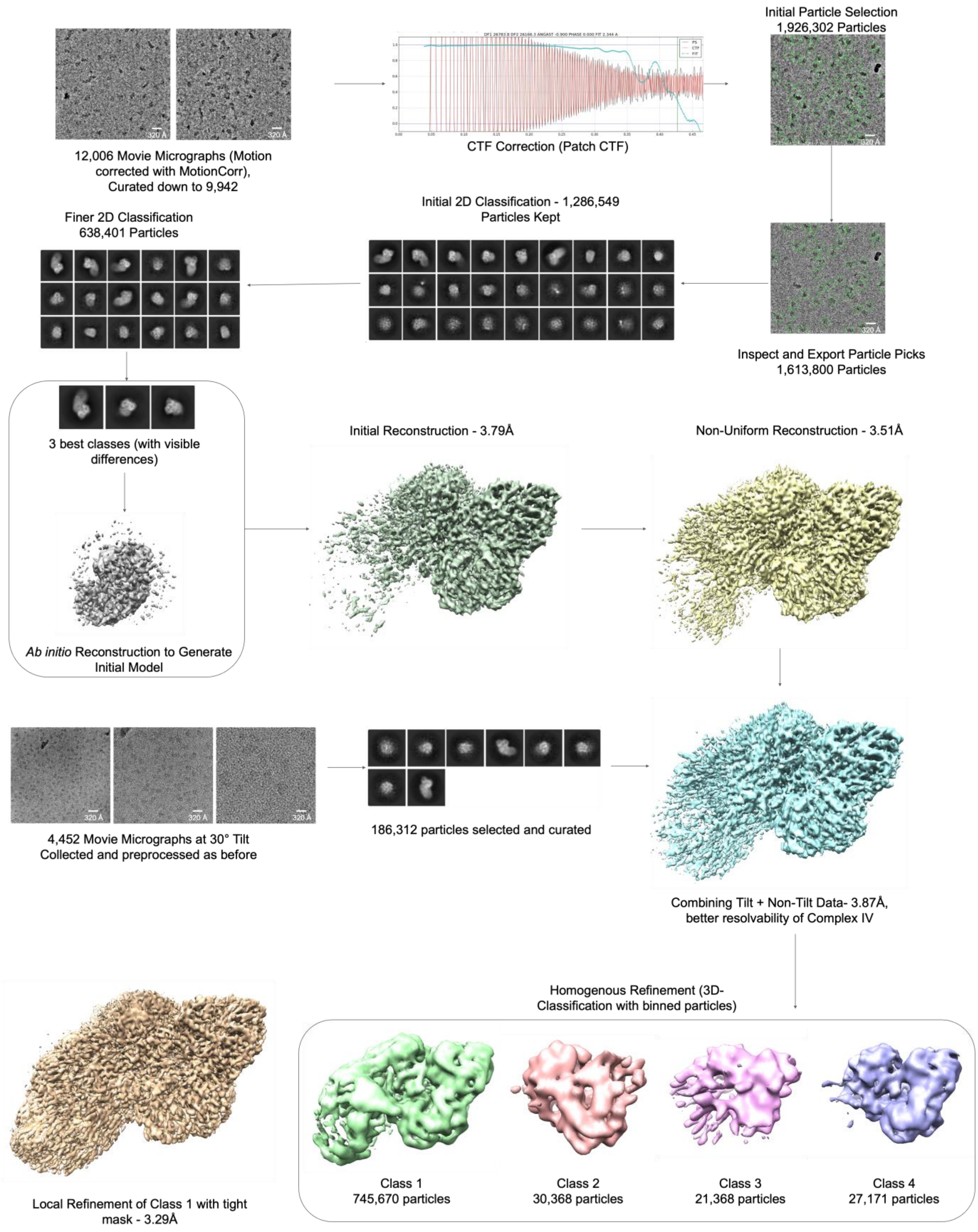

Supplementary Figure 13. CRD1Δ SC map refinement workflow. Note the addition of tilt data, which after 3D classification, improved the resolution and resolvability.

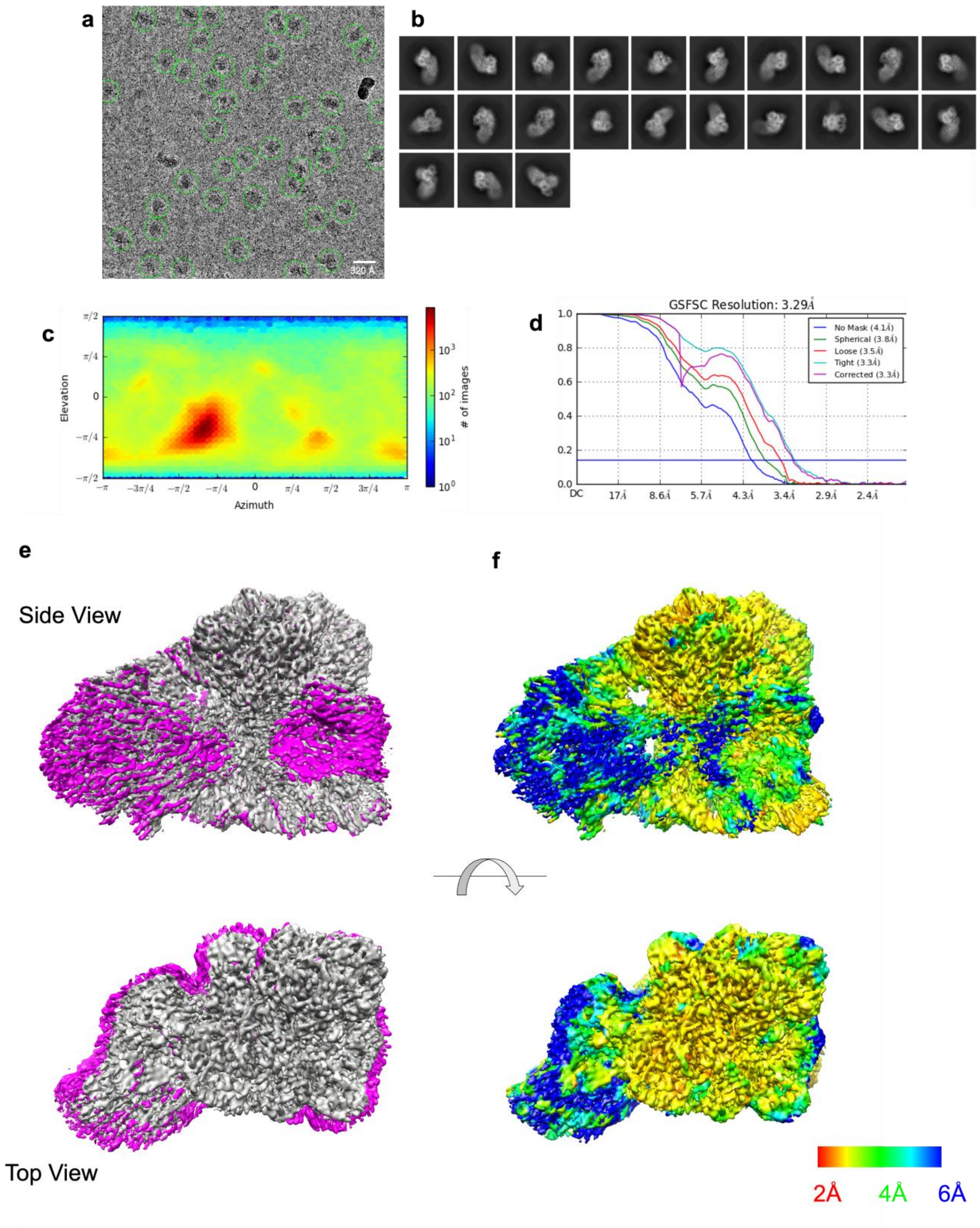

Supplementary Figure 14. Cryo-EM map details of the CRD1Δ SC. **a**. An example (out of scores of similar views) micrograph movie of the CRD1Δ SC (See Methods for additional details). **b**. Representative 2D class averages. **c**. This abundance of top views can be further displayed by assessing orientation determination. **d**. Fourier shell correlation plot revealing the resolution with various masks applied. **e**. The final cryo-EM density map (side and top views) is show with the excess detergent region (noise) displayed in magenta. This density was masked out after refinement leaving only the grey density, which was then modeled. **f**. Local resolution plot was mapped onto the structure (shown with a side and top view).

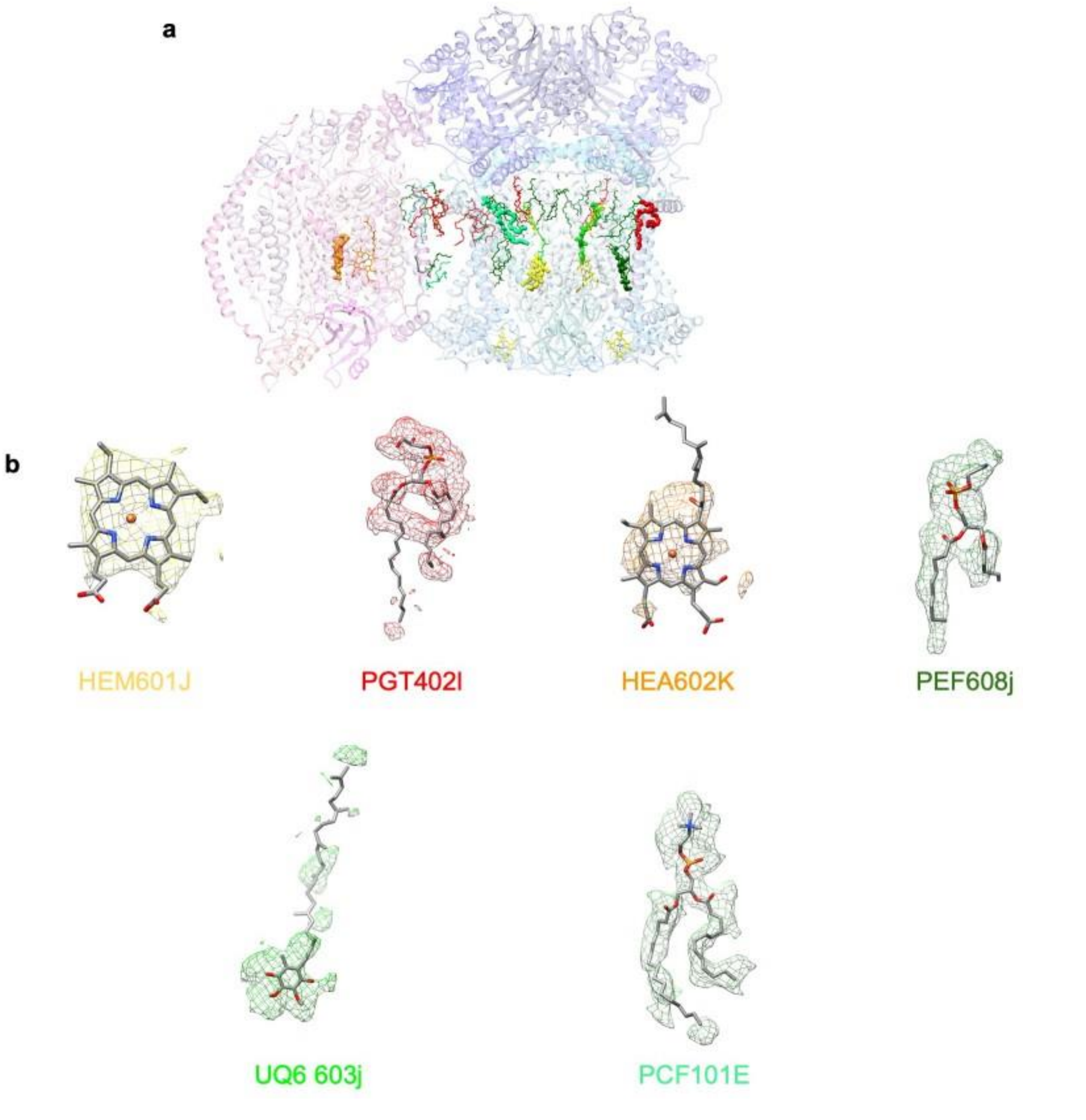

Supplementary Figure 15. Gallery of CRD1Δ SC ligands colored by type. (**a**) The CRD1Δ SC model is shown at the top with ligand densities colored for representative views. (**b**) Representative ligands (from panel **a**) are shown zoomed-in with the model and their isolated densities from the 3.3 Å map.

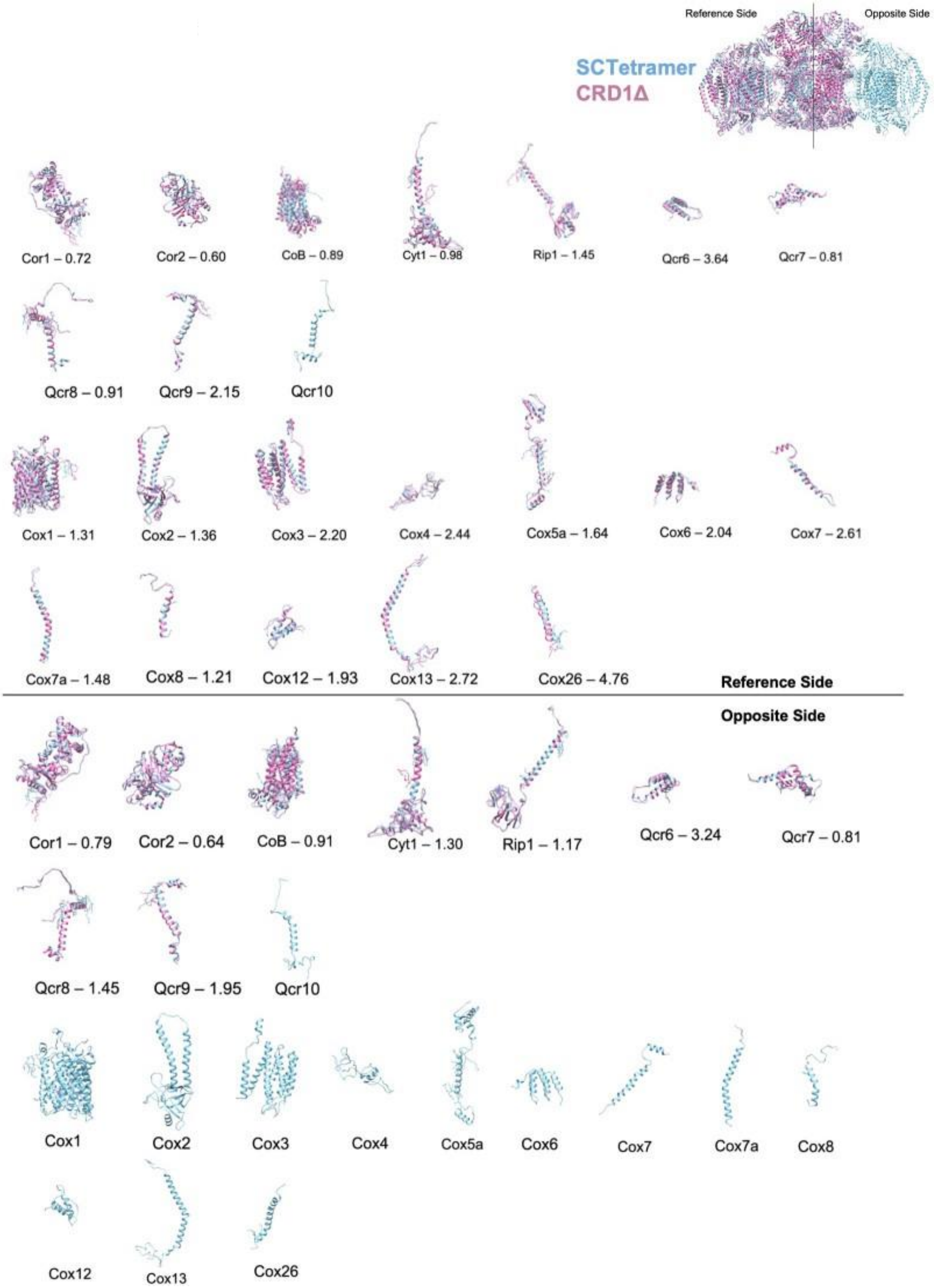

Supplementary Figure 16. Comparing individual chains in the WT SC tetramer and the CRD1Δ SC models. Each individual protein chain is shown for both structures (WT in light blue, CRD1Δ in pink). The CRD1Δ protein chains were aligned to the SC tetramer protein chains using Chimera MatchMaker tool. The RMSD was computed per-atom, across the whole protein chain, and the average value in Å is shown next to the protein name. Instances where no CRD1Δ SC protein was modeled are shown with only the tetramer protein and no value representing the RMSD. Subunits above the black line are associated with the reference-side and below are associated with the opposite side.

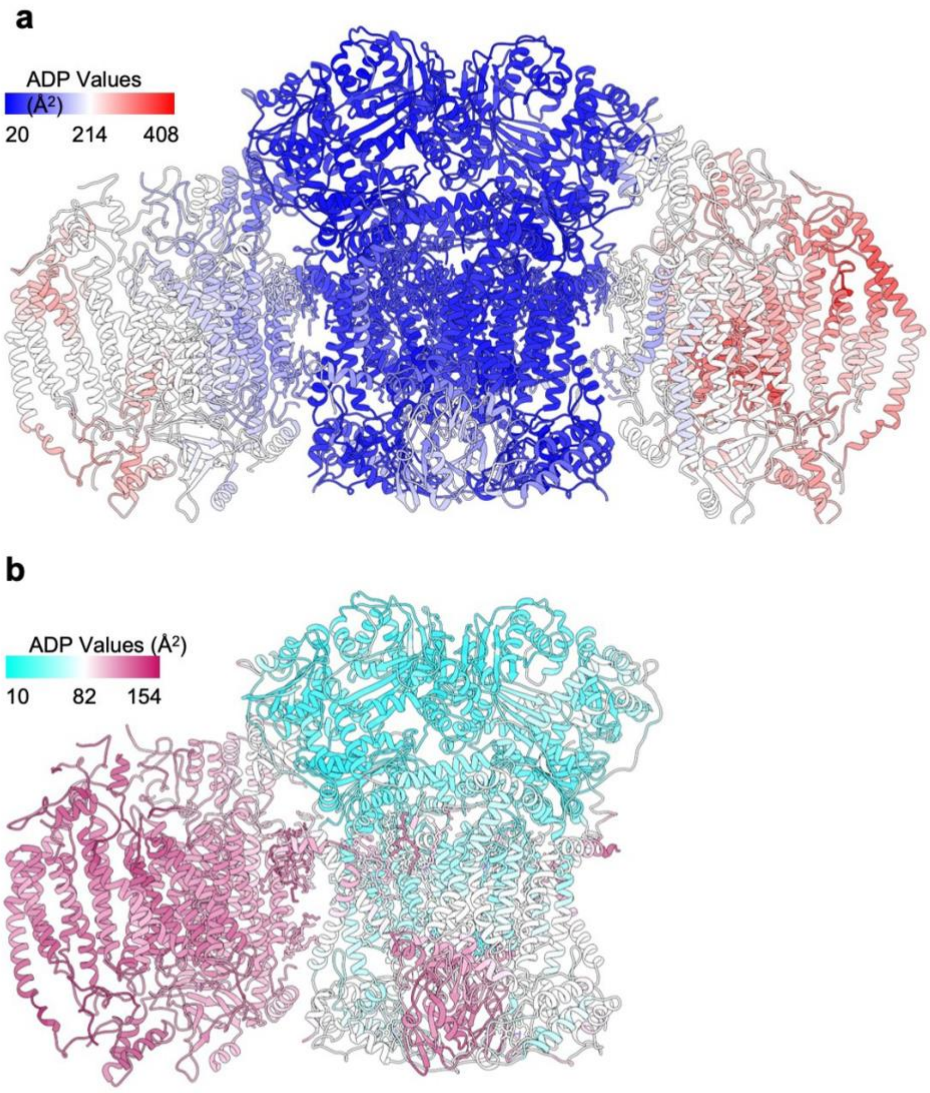

Supplementary Figure 17. Atomic displacement parameters. ADP values were computed during model refinement for (**a**) the WT SC tetramer and (**b**) CRD1Δ SC. These values were mapped onto the models to gain an understanding of modeled atom position uncertainty based on the experimental density map. During refinement, group B-factors were computed by Phenix, that is, one ADP value is optimized and assigned at the amino acid / ligand / cofactor level. It should be noted that B-factors were independently refined for the two structures, and thus exact ADP values should not be directly compared.

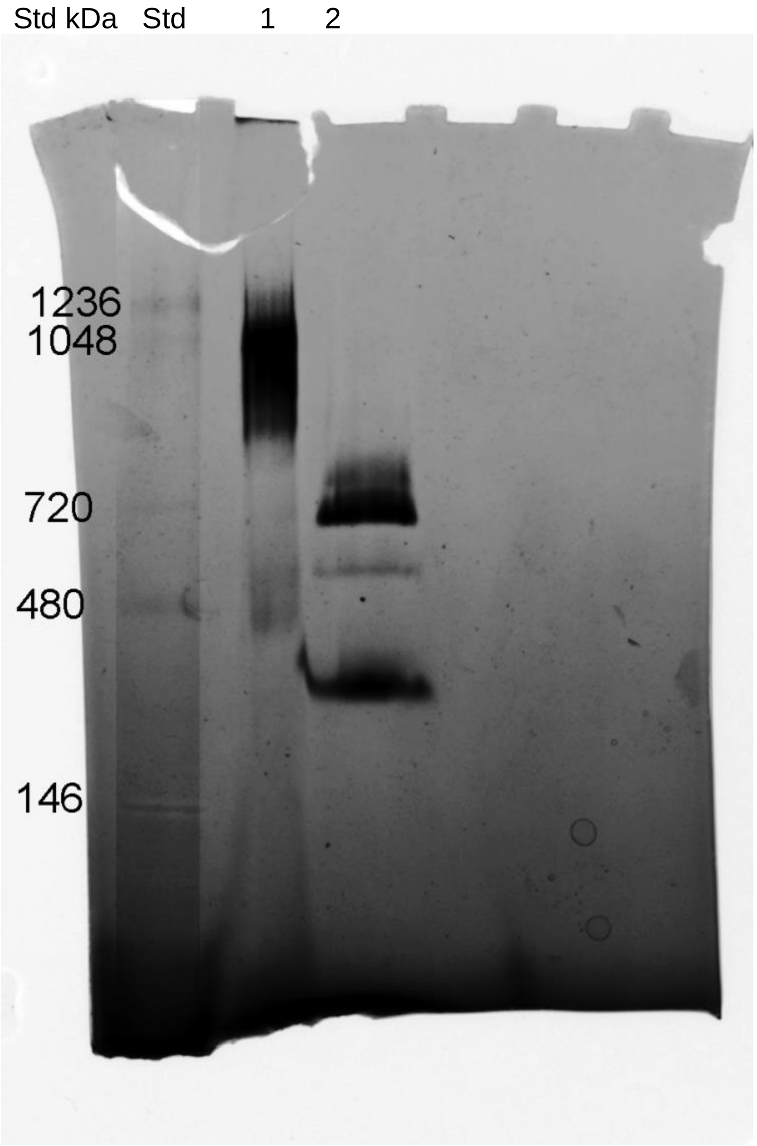

Supplementary Figure 18. Coomassie Blue stained gel from Supplementary Fig. 3c after in-gel assay for CIV. Lane 1 shows a single band from the purified WT SC (~1000 kDa). Lane 2 contains 3 bands from the purified ΔCRD1 SC (III_2IV_1 ~800 kDa, III_2 ~600 kDa, IV, ~220 kDa). The composition and molecular mass of these complexes were verified by Western blots using specific antibodies against CIII and CIV and cryo-EM data for the tetrameric and trimeric SCs containing their respective CIII and CIV. Native gel electrophoresis mobilities, especially for membrane proteins, are dependent on many variables that affect apparent molecular mass values and do not always conform to soluble protein MW standards shown on the left.

Supplementary Table 1. Data collection and map refinement

**Cryo-EM data collection, map refinement**	
**Data collection and processing**	
Magnification	130,000
Voltage (kV)	300
Electron Exposure ($e^-/Å^2/s$)	1.4 (total dose of 49$e^-/Å^2$ fractionated over 35 frames)
Defocus range (µm)	-1.5 to -3.5
Pixel size (Å)	1.07
**WT SC Tetramer**	
Symmetry imposed	C1, C2 to assess
Initial number of particles (no.)	1,510,025
Final number of particles (no.)	413,626
Map resolution (Å) at 0.143	3.19 (3.9Å with a loose mask)
Map Sharpening B factor (Å)	-116.8
**CRD1Δ Trimer**	
Symmetry imposed	C1
Initial number of particles (no.)	2,471,699 (non-tilt + tilt data)
Final number of particles (no.)	745,670
Map resolution (Å) at 0.143	3.29 (3.5Å with loose mask)
Map Sharpening B factor (Å)	-127.9

Supplementary Table 2. Model statistics and validation.

**Model Refinement**			
	**WT SC Tetramer**	**WT SC Trimer**	**CRD1∆ Trimer**
Initial model used (PDB Code)	6HU9	SC Tetramer (which started with 6HU9)	SC Tetramer (which started with 6HU9)
Nonhydrogen Atoms	62851 (64 Ligands)	48169 (48 Ligands)	46624 (46 Ligands)
ADP (B-factor) Range			
min/max/mean			
Protein	19.54/407.59/139.46	19.54/315.45/102.00	10.26/153.80/80.29
Ligand	34.11/379.08/110.82	34.11/253.91/86.27	35.72/152.76/91.57
**Model Validation**			
R.M.S. Deviation			
Bond lengths (Å)	0.004	0.005	0.003
Angles (°)	0.688	0.739	0.606
MolProbity score	2.21	2.24	2.04
Clash score	16.12	16.42	12.48
Poor Rotamers (%)	0.06	0.08	0.00
Ramachandran Plot			
Favored (%)	91.62	91.06	93.28
Allowed (%)	8.26	8.78	6.70
Outliers (%)	0.12	0.16	0.02
Map to Model FSC at 0.5	4.2	4.1	3.9

Supplementary Table 3. Representative calculation for oxygen consumption from graphs in Supplementary Figure 2. Chamber volume was 2 ml and protein concentrations are in mg/ml. Oxygen consumption was performed 3 times for all samples except for ∆CRD1 SC sample, which was done once. In the text O_2 consumption is converted to µmoles O_2/min/mg. See Methods for details. The software package associated with the instrument is DatLab-version 7.3.0.3. Source data are provided in the Source Data file.

WT Mitochondria

**Oxygen**	µM	**time**
Basal	212.8	3.0
+UQH2	153.8	
**Basal-UQH2**	58.9	
µM/min	19.6	
**Protein concentration**	**0.025**	
**Oxygen (µM/(min/mg))**	**785.94**	

∆*CRD1* Mitochondria

**Oxygen**	µM	**time**
Basal	221.5	6.0
+UQH2	171.6	
**Basal-UQH2**	49.8	
µM/min	8.3	
**Protein concentration**	**0.025**	
**Oxygen (µM/(min/mg))**	**332.22**	

WT SC

**Oxygen**	µM	**time**
Basal	243.9	0.9
+UQH2	211.5	
**Basal-UQH2**	32.4	
µM/min	36.0	
**Protein concentration**	**0.02**	
**Oxygen (µM/(min/mg))**	**1798.79**	

∆CRD1 SC

**Oxygen**	µM	**time**
Basal	262.2	3.0
+UQH2	246.3	
**Basal-UQH2**	15.9	
µM/min	5.3	
**Protein concentration**	**0.015**	
**Oxygen (µM/(min/mg))**	**353.88**	

Supplementary Table 4. Chain/Subunit IDs assigned for WT SC tetramer protein subunits, and associated ligands and ions. Assignments of associated ligands and ions are primarily based on distance calculated with phenix.sort_hetatms (https://phenix-online.org/documentation/reference/sort_hetatms.html) which matches all heteroatoms (ligands and ions) to the nearest polymer chain (which may or may not be involved in an interaction).

Chain ID	Protein Subunit	Associated Ligands and Ions
A,a	Cor1	A503PEF, a503PEF
B,b	Cor2	
C,c	Rip1	C301FES, C302CDL, C303PEF, c301FES, c302CDL, c303PEF
D,d	Qcr10	
E,e	Qcr9	E101PCF, e101PCF
F,f	Qcr7	
G,g	Qcr6	
H,h	Qcr8	H102PEF, H601CDL, H607PEF, h601CDL, h607PEF, h608PEF
J,j	CoB	J402PEF, J405CN5, J501CDL, J502PEF, J601HEM, J602HEM, J603UQ6, J604PEF, J605PEF, J606QU6, J608PEF j402PEF, j501CDL, j502PEF, j601HEM, j602HEM, j603UQ6, j604PEF, j605PEF, j606QU6, j608PEF
K,k	Cox1	K594CU, K601CDL, K602HEA, K603HEA k594CU, k601CDL, k602HEA, k603HEA
L,l	Cyt1	L401HEM, L402CDL, l401HEM, l402CDL
M,m	Cox8	
N,n	Cox7	
O,o	Cox3	
P,p	Cox2	P301CUA, P302CUA, p301CUA, p302CUA
Q,q	Cox6	
R,r	Cox7a	
S,s	Cox13	
T,t	Cox4	
U,u	Cox12	
V,v	Cox26	V607PEF, v607PEF
W,w	Cox5a	W201PEF, W202PCF, W402PEF, W606PCF w201PEF, w202PCF, w402PEF, w606PCF

Supplementary Table 5. Amounts in nmol/mg of protein of CL or PG determined using electrospray ionization mass spectrometry (see spectra in Supplementary Fig. 12) in WT and *CRD1*Δ mutant yeast mitoplasts. For each mass/charge (m/z) value and the sum of all the measured CL (Supplementary Fig. 12 **b** and **d**) or PG (Supplementary Fig. 12 **a** and **c**) are tabulated below. The values reported for CL from the mutant spectrum (**d**) fall within background levels. MS was only performed once so there are no statistics presented. Source data are provided in the Source Data file.

	m/z	Side Chain	Double Bonds	WT nmol/mg	CRD1Δ nmol/mg
**CL**	**1344**	64	4	0.5229	0.0039
	**1372**	66	4	1.0744	0.0091
	**1400**	68	4	1.6139	0.0391
	**1428**	70	4	1.5795	0.0370
	**1456**	72	4	0.9198	0.0197
	**Sum of CL Species**			**5.7105**	**0.1088**
**PG**	**691.6**	30	2	0.0023	0.3351
	**693.6**	30	1	0.0008	0.1034
	**717.6**	32	2	0.0186	0.5376
	**719.6**	32	1	0.0554	1.1099
	**745.6**	34	2	0.0554	0.9211
	**747.6**	34	1	0.1156	1.2283
	**773.6**	36	2	0.0326	0.4889
	**775.6**	36	1	0.0265	0.6431
	**Sum of PG Species**			**0.3072**	**5.3674**

Note: Although there are apparent trace peaks (within background levels) in the regions for singly ionized CL (Supplementary Fig. 12 **d**) in the *CRD1*Δ mutant spectrum, there are no corresponding CL doubly ionized peaks separated by 0.5 mass units, which would be a signature for the presence of CL Therefore, we concluded lack of detectible CL in the mutant strain.

Supplementary Table 6. Chain/Subunit IDs assigned for CRD1Δ SC trimer protein subunits, and associated ligands and ions. See Table 3 legend for details.

Chain ID	Protein Subunit	Associated Ligands and Ions
A,a	Cor1	
B,b	Cor2	
C,c	Rip1	C301FES, C302PGT<br>c301FES
E,e	Qcr9	E101PCF<br>e101PCF
F,f	Qcr7	
G,g	Qcr6	
H,h	Qcr8	H102PEF, H601PGT, H607PEF<br>h607PEF
J,j	CoB	J402PEF, J501PGT, J502PEF, J601HEM, J602HEM, J603UQ6, J604PEF, J605PEF, J608PEF<br>j402PEF, j501PGT, j502PGT, j601HEM, j602HEM, j603UQ6, j604PEF, j605PEF, j608PEF
K,k	Cox1	K594CU, K601PGT, K602HEA, K603HEA
L,l	Cyt1	L401HEM, L402PGT<br>l401HEM, l402PGT
M,m	Cox8	
N,n	Cox7	
O,o	Cox3	
P,p	Cox2	P301CUA, P302CUA
Q,q	Cox6	
R,r	Cox7a	
S,s	Cox13	
T,t	Cox4	
U,u	Cox12	
V,v	Cox26	V607PEF
W,w	Cox5a	W201PEF, W202PCF, W606PCF

Supplementary Table 7. CRD1Δ trimer cross-correlation between PGT site density and fit ligands.

**Modeled Ligand**	**Cross-correlation Fit**	**Percent difference when compared to PGT**
PGT	0.715	
CDL	0.641	13.9%
PCF	0.648	9.8%
PEF	0.644	10.4%
