# Supplementary material for: Structural insights into cardiolipin replacement by phosphatidylglycerol in a cardiolipin-lacking yeast respiratory supercomplex
Source: Nat Commun. 2023 May 15;14:2783. doi: 10.1038/s41467-023-38441-5 (PMC10185535; doi:10.1038/s41467-023-38441-5)
Supplement: Supplementary file 3 — Reporting Summary [file 41467_2023_38441_MOESM3_ESM.pdf]

nature portfolio

Corresponding author(s): William Dowhan, Eugenia Mileykovskaya, Matthew Baker

Last updated by author(s): 4/11/2023

Reporting Summary

Nature Portfolio wishes to improve the reproducibility of the work that we publish. This form provides structure for consistency and transparency in reporting. For further information on Nature Portfolio policies, see our Editorial Policies and the Editorial Policy Checklist.

Statistics

For all statistical analyses, confirm that the following items are present in the figure legend, table legend, main text, or Methods section.

n/a	Confirmed	
☐	☒	The exact sample size (*n*) for each experimental group/condition, given as a discrete number and unit of measurement
☐	☒	A statement on whether measurements were taken from distinct samples or whether the same sample was measured repeatedly
☒	☐	The statistical test(s) used AND whether they are one- or two-sided<br>*Only common tests should be described solely by name; describe more complex techniques in the Methods section.*
☒	☐	A description of all covariates tested
☒	☐	A description of any assumptions or corrections, such as tests of normality and adjustment for multiple comparisons
☒	☐	A full description of the statistical parameters including central tendency (e.g. means) or other basic estimates (e.g. regression coefficient) AND variation (e.g. standard deviation) or associated estimates of uncertainty (e.g. confidence intervals)
☒	☐	For null hypothesis testing, the test statistic (e.g. *F*, *t*, *r*) with confidence intervals, effect sizes, degrees of freedom and *P* value noted<br>*Give P values as exact values whenever suitable.*
☒	☐	For Bayesian analysis, information on the choice of priors and Markov chain Monte Carlo settings
☒	☐	For hierarchical and complex designs, identification of the appropriate level for tests and full reporting of outcomes
☒	☐	Estimates of effect sizes (e.g. Cohen's *d*, Pearson's *r*), indicating how they were calculated

Our web collection on statistics for biologists contains articles on many of the points above.

Software and code

Policy information about availability of computer code

Data collection	Titan Krios microscope (Thermo Fisher Inc.) equipped with EPU 2.10.0.5 software and post-GIF K2 Summit direct electron detector (Gatan), Oxygraph-2k high resolution respirometry system (Oroboros Instrument), API 4000 QTRAP (Sciex, Framingham, MA) mass spectrometer.
Data analysis	cryoSPARC v3.2, RELION3.0.8, EMAN2.31 and 2.91, Coot 0.9.5, MotionCor2_1.2.2, UCSF Chimera 1.16 and ChimeraX 1.2, Phenix 1.19.1-4122, pw_ligands.py v1.0, Ligplot+ v2.2, PDBePisa v1.52, GalaxyWeb (01/2019), Analyst v. 1.6.2, DatLab v. 7.3.0.3, MolProbity v. 4.5.2

For manuscripts utilizing custom algorithms or software that are central to the research but not yet described in published literature, software must be made available to editors and reviewers. We strongly encourage code deposition in a community repository (e.g. GitHub). See the Nature Portfolio guidelines for submitting code & software for further information.

Data

Policy information about availability of data

All manuscripts must include a data availability statement. This statement should provide the following information, where applicable:

- Accession codes, unique identifiers, or web links for publicly available datasets
- A description of any restrictions on data availability
- For clinical datasets or third party data, please ensure that the statement adheres to our policy

The EMDB IDs are EMD-27940 [https://www.ebi.ac.uk/emdb/search/EMD-27940] and EMD-28011 [https://www.ebi.ac.uk/emdb/search/EMD-28011] with their respective PDB IDs being 8E7S [https://www.rcsb.org/structure/unreleased/8E7S] and 8EC0 [https://www.rcsb.org/structure/unreleased/8EC0] for the WT and

CRD1Δ structures, respectively. The following pubic data based were used. 6HU9 [10.2210/pdb6HU9/pdb], 6YMX [10.2210/pdb6ymx/pdb], 1KB9 [10.2210/pdb1kb9/pdb], 3CX5 [10.2210/pdb3cx5/pdb], 6Q9E [10.2210/pdb6Q9E/pdb], 6GIQ [10.2210/pdb6Q9E/pdb] All data with no restrictions are available from the corresponding authors.

Human research participants

Policy information about studies involving human research participants and Sex and Gender in Research.

Reporting on sex and gender	N/A
Population characteristics	N/A
Recruitment	N/A
Ethics oversight	N/A

Note that full information on the approval of the study protocol must also be provided in the manuscript.

Field-specific reporting

Please select the one below that is the best fit for your research. If you are not sure, read the appropriate sections before making your selection.

☒ Life sciences ☐ Behavioural & social sciences ☐ Ecological, evolutionary & environmental sciences

For a reference copy of the document with all sections, see nature.com/documents/nr-reporting-summary-flat.pdf

Life sciences study design

All studies must disclose on these points even when the disclosure is negative.

Sample size	Micrographes containing individual particle images were acquired automatically using EPU on the Titan Krios cryo-TEM. For WT SC, a total of, a total of 1,510,025 particles were selected; after classification and refinement, the images were narrowed down to 413,626 particles.. For the ΔCRD1 dataset, 1,926,302 particles were selected from 12,006 micrographes and trimmed down to 638,401 particles. No statistical methods were used to determine EM data sample size. Biochemical assays were typically done in triplicate unless otherwise indicated. Statistical analysis was not used to determine sample size but was used to determine standard deviation.
Data exclusions	Iterative 2D and 3D classifications were used to eliminate particles. For all other experiments number of measurements made is listed and all gave similar results. For oxygen consumption experiments results during familiarization and initial setup of equipment were excluded.
Replication	Samples for cryo-EM studies were derived from one purification of the SCs from either the WT strain or the deltaCRD1 strain. Reproducibility of the final map was assessed using the gold-standard Fourier shell correlation at 0.143 - the data was randomly split into two halves, each half was processed independently and then compared, resulting in the final map at the reported resolution. Multiple rounds of refinements were carried out with variations to the image processing parameters. All resulting maps were nearly identical results. For gels and blots images reported were representative of multiple runs and in close agreement with previously published results by the authors. Numbers of experimental attempts for oxygen consumption measurements are reported and only a single mass spec analysis of each sample is reported.
Randomization	Randomization is implemented in image processing. Individual particle data sets were randomly split into odd and even halves and refined independently as noted above. For gels, blots, mass spectral measurements and assays multiple measurements (single if noted) were performed and grouped as to pertaining to WT or mutant strain samples. This study did not include experiments with experimental group allocation and thus no randomization was applied.
Blinding	No blinding of samples was performed and is not applicable in cryo-EM. Also each structure was derived from data accumulated on a single preparation of the tetrameric and trimeric complexes. This study did not include experiments with experimental group allocation and thus no blinding was applied. All experimental samples were prepared and analyzed as described in the manuscript.

Reporting for specific materials, systems and methods

We require information from authors about some types of materials, experimental systems and methods used in many studies. Here, indicate whether each material, system or method listed is relevant to your study. If you are not sure if a list item applies to your research, read the appropriate section before selecting a response.

Materials & experimental systems

n/a	Involved in the study
☐	☒ Antibodies
☒	☐ Eukaryotic cell lines
☒	☐ Palaeontology and archaeology
☒	☐ Animals and other organisms
☒	☐ Clinical data
☒	☐ Dual use research of concern

Methods

n/a	Involved in the study
☒	☐ ChIP-seq
☒	☐ Flow cytometry
☒	☐ MRI-based neuroimaging

Antibodies

Antibodies used	For CIII or CIV visualization custom polyclonal antibodies (prepared by Cocalico Biologicals) against purified CIII (raised in rabbits, 0.46 mg/m, diluted 1:2,500) and purified CIV (raised in guinea pigs, 0.77 mg/m/ diluted 1:5,000) were used. These antibodies were verified by detection of purified CIII and CIV as well as only complexes containing CIII or CIV in crude extracts. Secondary antibody peroxidase conjugated AffiniPure Goat anti-rabbit (cat. #111-035-003, diluted 1:10,000) and anti-Guinea pig IgG (H+L) (cat. #106-035-003, diluted 1:10,000) from Jackson ImmunoResearch Laboratories, Inc. were used.
Validation	CIII and CIV primary antibodies were verified by detection of purified CIII and CIV as well as only complexes containing CIII or CIV in crude extracts.
